# Granulocyte-CSF induced inflammation-associated cardiac thrombosis in iron loading mouse heart and can be attenuated by statin therapy

**DOI:** 10.1186/1423-0127-18-26

**Published:** 2011-04-15

**Authors:** Wei S Lian, Heng Lin, Winston TK Cheng, Tateki Kikuchi, Ching F Cheng

**Affiliations:** 1 Department of Medical Research, Tzu Chi General Hospital and Department of Pediatrics, Tzu Chi University, Hualien, Taiwan; 2 Institute of Biomedical Sciences, Academia Sinica, Taipei, Taiwan; 3 Department of Animal Science and Technology, National Taiwan University, Taiwan; 4 Institute of Toxicology and Pharmacology, Tzu Chi University, Hualien, Taiwan; 5 Department of Animal Science and Biotechnology, Tunghai University, Taichung, Taiwan

## Abstract

**Background:**

Granulocyte colony-stimulating factor (G-CSF), a hematopoietic cytokine, was recently used to treat patients of acute myocardial infarction with beneficial effect. However, controversy exists as some patients developed re-stenosis and worsened condition post G-CSF delivery. This study presents a new disease model to study G-CSF induced cardiac thrombosis and delineate its possible mechanism. We used iron loading to mimic condition of chronic cardiac dysfunction and apply G-CSF to mice to test our hypothesis.

**Methods and Results:**

Eleven out of fifteen iron and G-CSF treated mice (I+G) showed thrombi formation in the left ventricular chamber with impaired cardiac function. Histological analysis revealed endothelial fibrosis, increased macrophage infiltration and tissue factor expression in the I+G mice hearts. Simvastatin treatment to I+G mice attenuated their cardiac apoptosis, iron deposition, and abrogated thrombus formation by attenuating systemic inflammation and leukocytosis, which was likely due to the activation of pAKT activation. However, thrombosis in I+G mice could not be suppressed by platelet receptor inhibitor, tirofiban.

**Conclusions:**

Our disease model demonstrated that G-CSF induces cardiac thrombosis through an inflammation-thrombosis interaction and this can be attenuated via statin therapy. Present study provides a mechanism and potential therapy for G-CSF induced cardiac thrombosis.

## Background

Granulocyte colony-stimulating factor (G-CSF), a hematopoietic cytokine, induces mobilization of the hematopoietic stem cells from the bone marrow into the peripheral blood circulation. In traditional bone marrow transplantation, G-CSF is given to healthy donors for allogenic hematopoietic cell collection [1,2]. Recently, G-CSF has been used to treat acute myocardial infarction (AMI) patients with intention to mobilize autologous stem cells and thus to replace infarct cardiac muscle cells. Although G-CSF treatment improved cardiac function in both clinical studies and in animal models of AMI [3-5], this treatment remains controversial since equivocal benefits [6-8] and some AMI patients developed re-stenosis and worsened condition post G-CSF delivery [9,10]. In addition, three cases of late stent thrombosis were reported in a cohort study of 24 patients who had undergone intra-coronary infusion of G-CSF after primary stenting for AMI [11]. These observations raise concerns about the clinical long-term safety profile of G-CSF therapy for AMI patients. It is suggested that G-CSF may induce a hyper-coagulable state due to the combination of activated endothelial cells and increased platelet-neutrophil complex formation [12-14]. However, the type of patients that are at risk for thrombosis as well as the mechanism underlying G-CSF related thrombosis is still not clear.

In the present study, a new *in vivo *disease model to study G-CSF induced cardiac thrombosis in mice is presented. We assumed that patients with atherosclerosis, diabetes, chronic heart failure, or other diseases with chronic inflammation or vasculopathy may be at higher risk for thrombosis after G-CSF treatment. Since chronic iron loading increases vascular oxidative stress and accelerate atherosclerosis [15-17]; we provided iron loading and G-CSF to mice to test our hypothesis by examining the incidence of cardiovascular thrombosis. Interestingly, intra-cardiac thrombus formation was observed in iron and G-CSF (I+G) treated mice. In addition, we showed that HMG-CoA reductase inhibitor, or statin therapy, could abrogate thrombus formation in I+G mice [18,19]. Using this novel animal disease model, our objective was to elucidate the molecular mechanism of post G-CSF cardiac thrombosis and to investigate possible modalities for its treatment and prevention.

## Materials and methods

### Mobilization of autologous stem cells by G-CSF

In order to test whether G-CSF can mobilize autologous stem cells, we divided male C57BL/6 mice (bw 25-30 gm) into four groups (n = 5/group) and injected them with 50, 100, 200 μg/kg bw G-CSF or saline daily for 5 days respectively. Blood serum was then harvested for flow analysis.

### Iron loading and G-CSF administration

**Figure 1:**
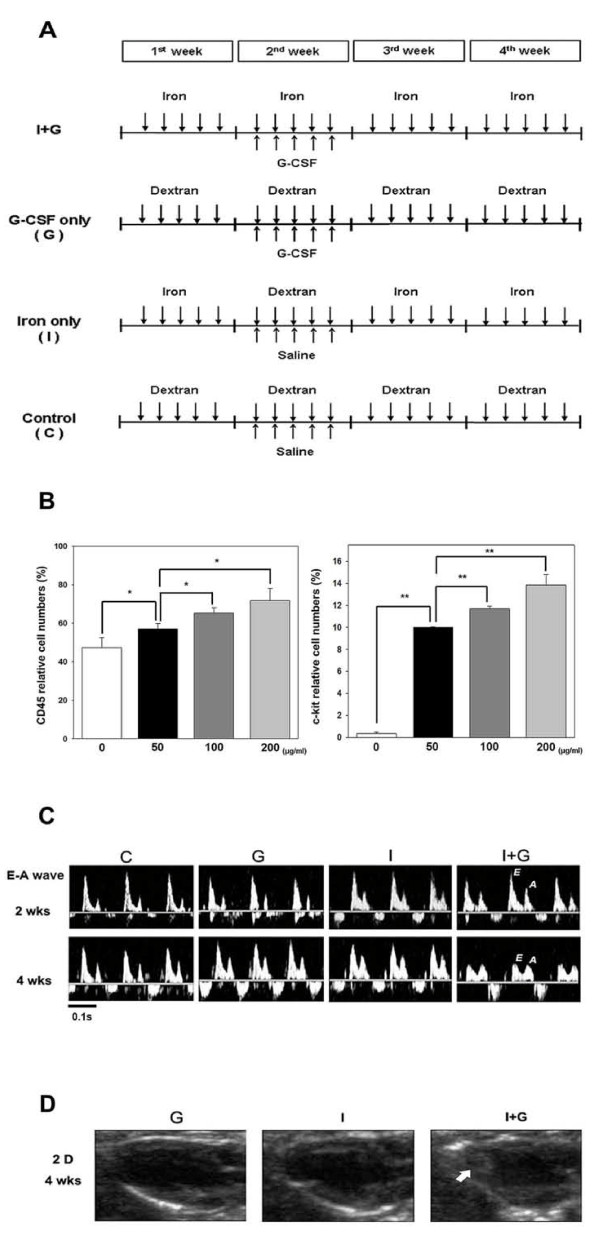
**Protocols using G-CSF to mobilize stem cells and echocardiographic assessment of cardiac function in mice**. (A) Animals were divided into four groups for 4 weeks of iron intra- peritoneally injection (10 mg/25 gm body weight of mouse per day for 5 days/week) or dextran injection as shown in the protocols. G-CSF (100 μg/kg/day subcutaneous injection) or saline was given for 5 days in the second week as shown. I+G; iron plus G-CSF treatment. (B) Different dosages of G-CSF were given to mice with blood c-kit and CD45 examined by flow cytometry analysis. (C) Representative echocardiograms of mitral-valve-flows Doppler mapping (E and A waves) in each experimental group at end of the second and fourth week, respectively. Decreased E: A wave ratio showing diastolic dysfunction in the I+G group. E wave and A wave, indicating LV early-filling wave and filling from atrial contraction, respectively. (D) Representative 2D echocardiogram of long axis view revealed intra-cardiac mass (arrow) in the apex region of the left ventricle in the I+G group at 4^th ^week exam.

Male C57BL/6 mice (body weight (bw): 25-30 gm) were divided into four experimental groups (n = 15-18/group). (1) Iron loading and G-CSF supplement (I+G group): 10 mg/25 gm bw/day iron dextran (Sigma-Aldrich Co. U.S.A.), was injected five times/week intraperitoneally (ip) for 4 weeks, and 100 μg/kg bw recombinant human G-CSF (Granocyte, Chugai Pharmaceutical, Co., Ltd, Tokyo, Japan), was administered five times/week subcutaneously during the second week. (2) G group: Dextrose (0.1 ml of 10%) instead of iron dextran was injected five times/week for 4 weeks. G-CSF was administered as in I+G group. (3) I group: 0.1 ml saline (instead of G-CSF) was administered subcutaneously five times/week during the second week and iron dextran was injected as I+G group. (4) Control or C group: Only 10% dextrose and saline solutions were administered as in I+G group (Figure 1A). Mice underwent *in vivo *cardiac echocardiography at the end of the second and fourth week. Similar protocols of iron loading and G-CSF supplement to mice were previously described [3,20].

### Simvastatin or tirofiban treatment to I+G mice, blood counts and serum ELISA

The second set of male C57BL/6 mice were injected (ip) with 10 mg/kg bw simvastatin (USP, Laucala Campus Suva, Fiji Islands) for first two weeks (days 1^st^, 3^rd^, and 5^th^/week) in addition to four weeks of I+G treatment. Mice were divided into the following four groups (n = 10/group), I+G group, I+G plus simvastatin group (I+G+St), iron only group (I), and control or C. Protocols for iron loading and G-CSF supplement were the same as before. A third set of male C57BL/6 mice were injected with tirofiban (400 ug/kg, Merck & Co., INC.) using Alzet minipumps (model 2004, Alzet) for the first two weeks in addition to four weeks of I+G treatment. Mice were divided into the following three groups (n = 10/group), I+G group, I+G plus tirofiban group, and control group. Complete blood counts and leukocyte classification were checked with the CELL-DYN^® ^3700 (Abbott Park, Illinois, U.S.A.) and serum C-reactive protein (CRP, Immuno-Biological Laboratories, IBL, USA), ICAM-1 and MCP-1 level were determined with the Quantikine^® ^ELISA (R&D systems, Germany) using an ELISA plate reader at 450 nm with a correction at 570 nm.

### Echocardiography studies

Mice were anesthetized with pentobarbital (50 mg/kg body weight, ip). The anterior chest was shaved and laid in a left decubitous position with application of gel on the chest wall for better scanhead-skin contact. The echocardiography system (HDI 5000, Phllips, U.S.A.) was equipped with 2D, M-mode, and pulse wave Doppler imaging. Heart rate, left-ventricle (LV) dimension in both systolic and diastolic stages, the LV fractional shortening/ejection fraction and mitral valvular inflow with diastolic E and A waves in Doppler flow mapping were measured.

### Histology

Mice were perfused through the LV with 4% paraformaldehyde in 0.1 M PBS. The paraffin-embedded cardiac cross sections (5 μm) were stained with Hematoxylin & Eosin, Masson's trichrome and iron-specific-Prussian blue. Trichrome-stained sections were used to detect a cumulative index of myocardial damage, including fibrosis and inflammation. The cardiac coronary artery and liver paraffin section were stained with Hematoxylin & Eosin.

### Immunohistochemistry and immunofluorescent analysis

Mice were perfused transcardially with 4% paraformaldehyde in 0.1 M PBS and post fixed with the same fixative overnight at 4°C. Coronal heart were paraffin-embedded and tissue sections were cut into 5 μm thickness. After blocking deparaffinized sections and then treated with epitope retrieval buffer (Thermal scientific, Inc.) in 95~100°C for 30 min, and then quenched with 30% H_2_O_2 _and blocking 5% fetal bovine serum. The sections were then incubated with first antibody with rabbit anti-tissue factor (Santa Cruz, FL-295, 1:300), mouse anti-8-OHdG (Santa Cruz, 1:200), mouse anti-HNEJ-2 (Abcam, 1:200), mouse anti-CD45 (Thermo scientific, 1:200) and mouse anti-CD34 (Abcam, 1:150). Thereafter treated with a 1:200 dilution of biotinylated anti-mouse and anti-rabbit IgG antibody (KPL, Europe), followed by horseradish peroxidase (HRP)-conjugated streptavidin-biotin complex (Vectastain Elite ABC kit standard) for 1 hour at room temperature and then used 3,3-diaminobenzidine (DAB) as a chromogen (Vector Laboratories, Burlingame, CA), and counterstained with Contrast GREEN Solution (KPL, U.S.A.) for microscopic studies.

For immunofluorescent staining, sections were first rehydrated and epitope retrieval buffer (Thermal scientific, Inc.) in 95~100°C for 30 min. Sections were then washed and blocked with 5% fetal bovine serum for 1 hr. Sections were then double-stained with antibodies against TF (M-20, 1:100) and CD13 (1:100) overnight at 4°C. Different Fluorescein (FITC, donkey anti goat) and Rhodamine (TRITC, donkey anti rabbit) secondary antibodies (Jackson ImmunoResearch Lab. Inc.) were used to obtain fluorescent colors. The stained sections were counterstained with DAPI to visualize nuclei by ProLong antifade (Invitrogen) mounting reagent.

### Flow Cytometry Analysis

Flow cytometry analysis was performed with FACSCalibur and CellQuest Pro software (Becton Dickinson, San Joes, CA, USA) using directly conjugated mAbs against the following markers: CD11b-PE and Ly-6G-FITC or CD45-PE and CD117-PE (c-kit) (BD biosciences) with corresponding isotype matched controls. Blood samples were washed with PBS buffer and red blood cells were removed by RBC lysis buffer. Briefly, mAbs and cells were incubated for 30 minutes at 4°C and unbound reagents were removed by washing. Cells were then resuspended in fixing buffer (PBS containing 1%formaldehyde and 1% FBS) for flow analysis.

### RNA isolation and real-time PCR

Assays were performed using Applied Biosystems PRISM 7700 sequence detection system with cDNAs derived from mice treated with or without G-CSF following iron injection. Glyceraldehyde-3-phosphate dehydrogenase (GAPDH) was used as control. Thermal cycler conditions were as follows: hold for 2 min at 50°C and 10 min at 95°C, followed by two-step PCR for 35 cycles of 95°C for 15 s, then 60°C for 1 min. Forward and reverse primers and a fluorescence-labeled probe were as follows: ICAM-1 sense, 5'- CGC AAG TCC AAT TCA CAC TGA -3', and antisense, 5'- ATT TCA GAG TCT GCT GAG AC -3); MCP-1 sense, 5'- CAG CCA GAT GCA GTT AAC GC -3', and antisense, 5'- GCC TAC TCA TTG GGA TCA TCT TG -3'); tissue factor sense, 5'- AAG GAT GTG ACC TGG GCC TAT GAA -3', and antisense, 5'- ACT GCT GAA TTA CTG GCT GTC CGA T-3'); TNF-α sense, 5'- TAC TGA ACT TCG GGG TGA TTG GTC C -3', and antisense, 5'- GGT TCT CTT CAA GGG ACA AGG CTG -3') and GAPDH sense, 5'-GGA GCC AAA CGG GTC ATC ATC TC-3', and antisense, 5'-GAG GGG CCA TCC ACA GTC TTC T-3'). The relative expression ratio of each transcript (ICAM-1, MCP-1, tissue factor, and TNF-α) in comparison to GAPDH was calculated as described.

### Western blot analysis

Myocardium protein extracts were prepared by using a protein extraction kit (NE-PER), and total protein concentrations was determined by BCA™ protein assay reagent. Western Blot chemiluminescence reagents were obtained from PIERCE (Pierce Chemical Co.). Proteins were separated by polyacrylamide gel electrophoresis and transferred to PVDF membranes for Western blot analysis. Blots were incubated with either anti-p-AKT (1:1000), anti-AKT (1:1000), anti-eNOS (1:1000) (Cell Signaling Technology Inc.), anti-MPO (1:500) (R&D systems, Inc.) and anti-β-actin (1:2000) antibodies in non-fat dry milk in wash buffer overnight at 4°C. Blots were then incubated with peroxidase conjugated anti-rabbit (1:10,000) or anti-goat (1:1,000) for 1 hour at room temperature. Proteins were visualized by enhanced chemiluminescence, immunoblot signals were quantitated using a Fujifilm Medical Systems U.S.A., Inc.

### Statistical analysis

Statistical analysis was done by SPSS for Windows (version 12.0). All data are described as means ± standard deviation (S.D.). The two groups were compared using the Student's *t*-test. Statistical analysis was performed with one-way ANOVA by Tukey test for multiple comparisons. The differences were considered significant at a value of *P *< 0.05.

## Results

### G-CSF can mobilize autologous stem cell and effect cardiac dysfunction with intra-cardiac thrombosis in I+G mice

**Figure 2:**
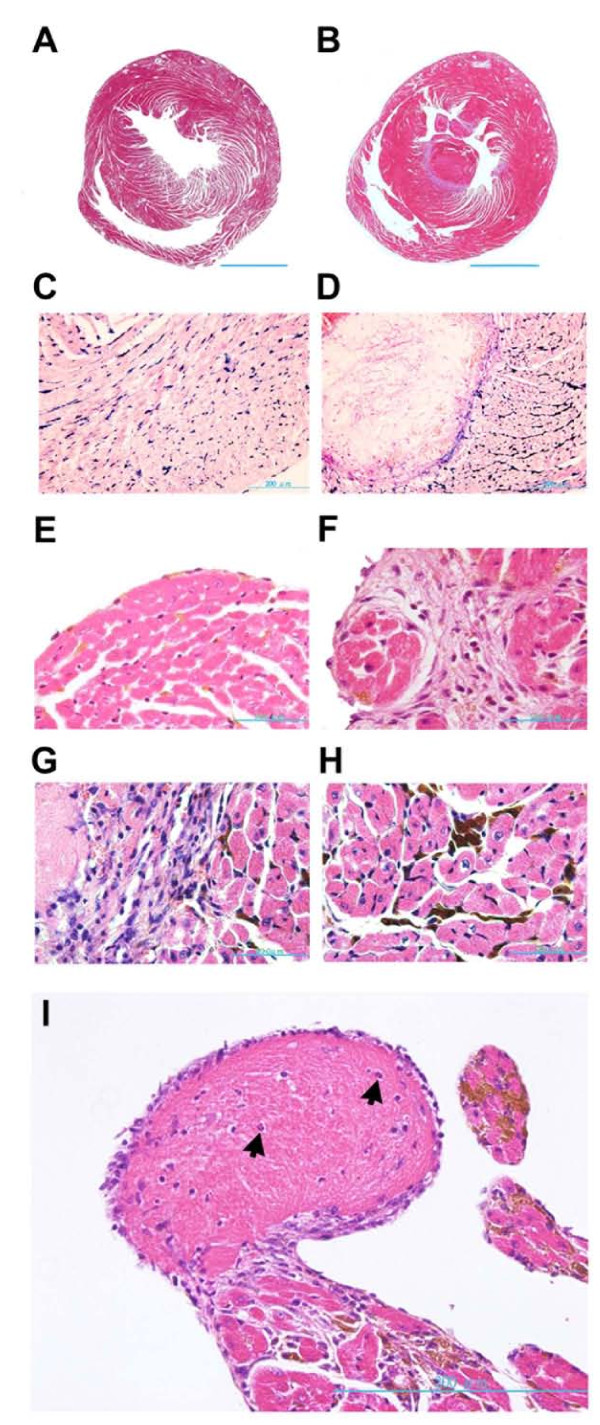
**Intra-cardiac thrombus formation and histopathology of the ventricular tissue in I+G heart**. (A and B) Heart cross-section at the papillary muscle level of the LV from iron (I) only (A and C) and I+G heart (B and D) stained with Masson's trichrome and Prussian blue staining, respectively. Note that the formation of a large mural thrombus in I+G heart. (E and F) Obvious fibrosis near the endocardium was noted in the I+G heart (F), but not in the iron only group (E). (G and H) Higher magnification of the LV from I+G group depicted regions of prominent fibrosis between thrombus and myocardium (G) and macrophages with cytoplasmic iron (brown color) deposition, infiltrated into intra-cardiomyocytic spaces (H). (I) Magnification of thrombus near the LV papillary muscle demonstrated leukocytes (arrows) involved in thrombus formation. Tissue section in E was stained with iron staining; tissue section in F, G, H, and I were stained with H & E staining.

We first used flow cytometry to check both c-kit(+) and CD45(+) cells from G-CSF injected mice to confirm that G-CSF can mobilize stem cells and leukocytes in a dosage dependent manner in our mice model before analyzing any phenotype (Figure 1B). Echocardiography at the end of 4^th ^week showed that heart functions in the I+G group was abnormal with decrement in fractional shortening and mild chamber dilation in the left ventricle (LV) without affecting the heart rate (Table 1). In addition, diastolic impairment was also found in the I+G group, with decreased E/A ratio progressively from the 2^nd ^to 4^th ^week (Figure 1C, Table 1). Interestingly, intra-cardiac thrombus were found in the LV at the 4^th ^week check up in I+G group (11/15 mice, Figure 1D). Histological examination by Masson trichrome staining confirmed the presence of intra-cardiac thrombus with fibrosis only in the I+G but not in other groups (Figures 2A and 2B).

**Table 1:** Echocardiographic results at the end of 2^nd ^and 4^th ^week in I+G and other experimental groups

	HR (bpm)	LVPWs (cm)	LVIDSs (cm)	IVSs (cm)	LVPWd (cm)	LVIDd (cm)	IVSd (cm)	EF (%)	FS (%)	E/A ratio
2wks										
C	360.5 ± 33	0.08 ± 0.01	0.22 ± 0.03	0.11 ± 0.01	0.07 ± 0.01	0.35 ± 0.03	0.06 ± 0.01	75.75 ± 5.1	37.90 ± 4.4	1.83 ± 0.22
G	333.0 ± 40	0.10 ± 0.02	0.25 ± 0.04	0.12 ± 0.01	0.07 ± 0.01	0.37 ± 0.02	0.07 ± 0.01	70.40 ± 11	33.53 ± 8.0	1.85 ± 0.23
I	372.2 ± 45	0.08 ± 0.02	0.24 ± 0.02	0.11 ± 0.02	0.05 ± 0.01	0.36 ± 0.04	0.06 ± 0.01	69.88 ± 3.6	33.50 ± 1.5	2.07 ± 0.59
I+G	362.9 ± 12	0.08 ± 0.01	0.24 ± 0.02	0.12 ± 0.02	0.06 ± 0.01	0.36 ± 0.02	0.06 ± 0.01	71.78 ± 5.6	35.23 ± 2.1	1.94 ± 0.39

4wks										
C	333.5 ± 78	0.10 ± 0.02	0.22 ± 0.04	0.11 ± 0.02	0.07 ± 0.01	0.35 ± 0.03	0.06 ± 0.01	73.68 ± 6.5	36.39 ± 5.4	1.89 ± 0.17
G	348.2 ± 32	0.08 ± 0.02	0.25 ± 0.02	0.10 ± 0.01	0.06 ± 0.01	0.36 ± 0.02	0.06 ± 0.01	65.58 ± 4.3	30.78 ± 2.6	1.85 ± 0.23
I	325.8 ± 95	0.08 ± 0.04	0.23 ± 0.06	0.10 ± 0.02	0.06 ± 0.03	0.34 ± 0.04	0.06 ± 0.02	68.50 ± 12.7	32.94 ± 11.3	1.97 ± 0.14
I+G	315.9 ± 58	0.09 ± 0.01	0.28 ± 0.02*	0.12 ± 0.01	0.06 ± 0.01	0.38 ± 0.02*	0.08 ± 0.01^†^	58.65 ± 4.5^†^	26.26 ± 2.8^†^	1.85 ± 0.22^†^

IVSd, inter-ventricular septum thickness at diastole; LVIDd, left ventricular internal diameter at diastole; LVPWd, left ventricular posterior wall thickness at diastole; IVSs, inter-ventricular septum thickness at systole; LVIDs, left ventricular internal diameter at systole; LVPWs, left ventricular posterior wall thickness at systole; FS, fractional shortening of left ventricle; EF, ejection fraction of left ventricle; E/A, E wave/A wave ratio at left ventricular diastolic phase;*p < 0.05, ^†^p < 0.01 vs control, n = 12 in each group.

### Cardiac histopathology of I+G mice

The mural thrombi found in I+G mice were mainly located in the apex region of the LV (Figure 1D), but also found in the chorda tendini of the LV (Figures 2B and 2I) and in the right ventricular cavity (data not shown). Histological analysis of the hearts from I group and I+G groups revealed iron deposition (Figures 2C and 2D). However, only I+G hearts revealed interstitial fibrosis with mural thrombi, attached tightly to the endocardium (Figures 2B and 2D). Extensive fibrosis was observed along the border between the cardiac endothelium and thrombi mass (Figure 2G). Macrophages with iron deposition in the cytoplasm infiltrated into the inter-myocytic spaces of the ventricular heart tissue (Figure 2H) and leukocytes were involved in thrombus formation (Figure 2I). However, there are no signs of thrombi formation in any body organs (aorta, liver, kidney and coronary arteries) examined (see Additional file 1, Figure S1).

### Increased expression of tissue factor in the I and I+G hearts and its co-localization with macrophage marker CD13

**Figure 3:**
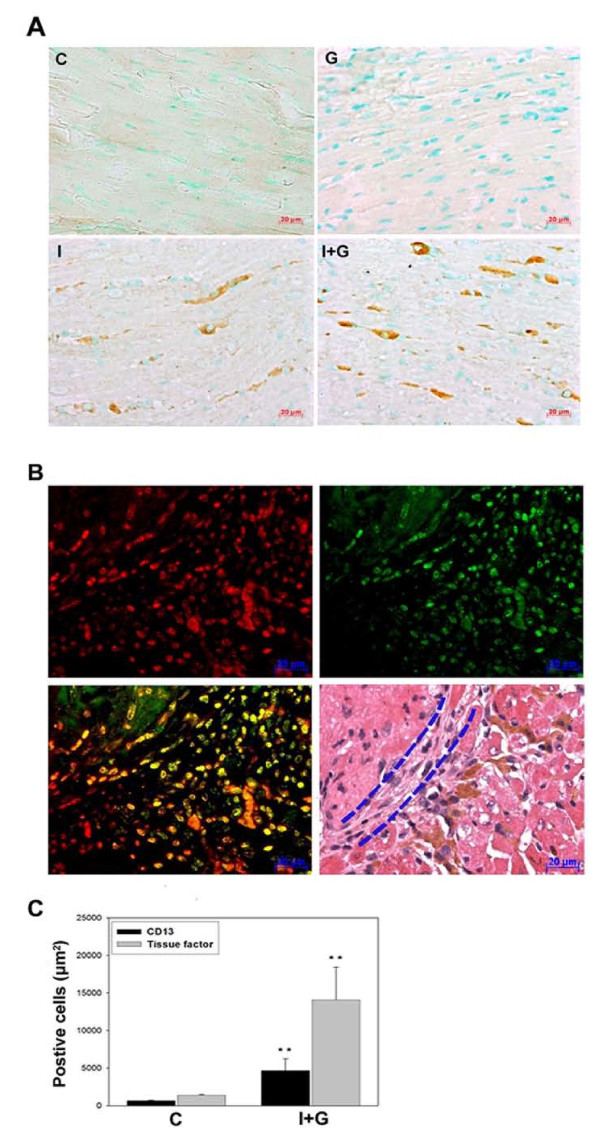
**Immunohistochemical detection of tissue factor and its colocalization with macrophage marker (CD13) in I and I+G hearts**. (A) Immunoreactivity of tissue factor was shown in I and I+G hearts, with more prominent in the latter group. (B) Colocalization of CD13 specific for monocytes/macrophage and tissue factor in heart tissue of I+G mice. Heart sections were stained with anti-tissue factor antibody (red in left upper panel), anti-CD-13 antibody (green in right upper panel), merge (left lower panel), and H & E staining (right lower panel). Co-localization of CD13 and tissue factor expression was seen in cardiac tissue near the heavy fibrosis region, implying region of prominent inflammation. Dashed line (in sections with H & E staining) indicated region of endocardium with cardiac fibrosis seen between thrombus (left upper) and myocardium (right lower). (C) Quantitative analysis of either tissue factor or CD13 staining positive cells in both control (C) and I+G hearts were shown in diagrams, **P < 0.001 vs control.

Cellular compositions of the all groups were examined by immunohistochemistry. Tissue factor was upregulated within the myocardium where it may be mediated by the infiltrating cells in both I and I+G groups, with more prominent in the latter group (Figure 3A). Confocal microscopy depicted colocalization of CD13 (a protein specific for monocytes/macrophages) with tissue factor near the endocardium-myocardium junction in the I+G heart tissue, implying areas of prominent inflammation (Figure 3B). Here we demonstrated that G-CSF enhances the recruitment of monocytes/macrophages and the expression of tissue factor in the affected heart tissue especially in the I+G group (Figure 3C).

### G-CSF supplement aggravates iron induced oxidative stress, leukocyte infiltration and inflammatory profile in heart

**Figure 4:**
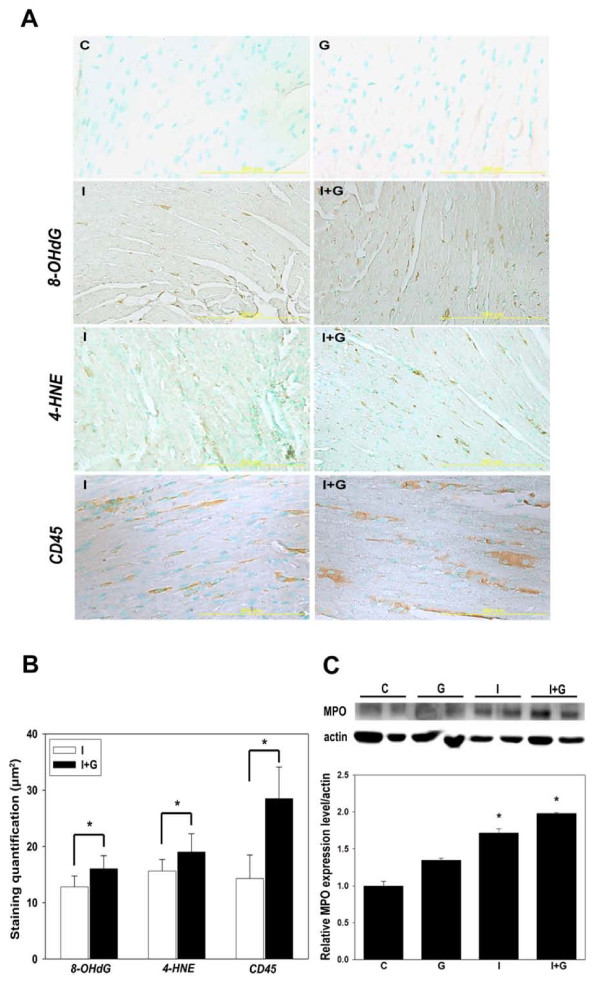
**G-CSF enhanced iron induced oxidative stress and leukocyte infiltration with aggravation of myeloperoxidase (MPO) activity in heart**. (A and B) Immunoreactivity of 8-OHdG, 4-HNE (both are markers for oxidative stress) and CD45 (leukocyte marker) were compared and quantified between iron only (I) and I+G heart tissue. Representative results of three separate experiments are shown in (B). (C) MPO activities in heart tissue from all groups and their relative expression compared with actin were shown, *p < 0.01.

In order to elucidate the role of G-CSF in our I+G model, we compared the heart tissue from both I group and I+G group for oxidative stress, leukocyte infiltration and inflammatory profile between them. As expected, I+G hearts had higher levels of 4-HNE and 8-OHdG (both are index of oxidative stress), and increased expression of CD45 (leukocyte marker) (Figures 4A and 4B). Myeloperoxidase activity was also higher in the I+G hearts, indicating aggravation of inflammatory profile in the I+G hearts, as compared to the hearts from I group (Figure 4C).

### Simvastatin attenuates cardiac apoptosis, iron deposition, and thrombosis in I+G mice in vivo

**Figure 5:**
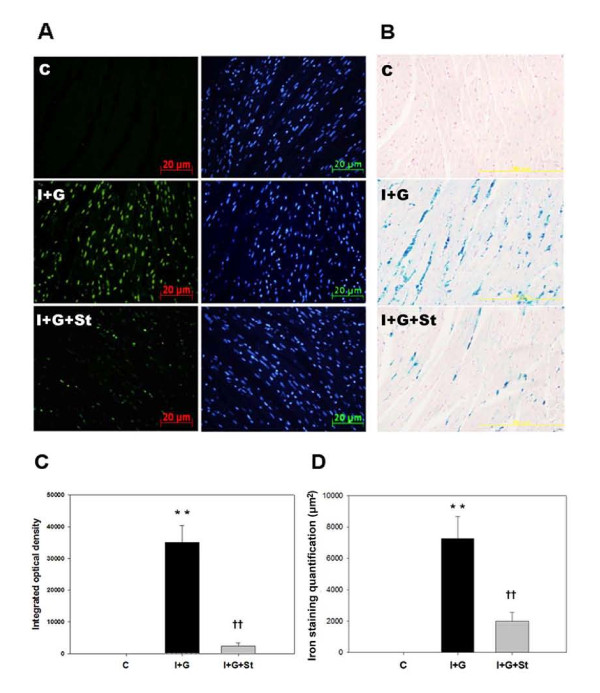
**Apoptosis and iron deposition/infiltration of cardiomyocytes following simvastatin treatment in I+G mice**. (A and C) Apoptotic cardiac myocytes were detected by the TUNEL assay in control group, I+G group, and I+G with simvastatin (I+G+St) treatment group respectively. Left and right panels show the TUNEL positive (green) and nuclei (blue) fluorescence, respectively. Each histogram represents the number of TUNEL-positive cells in each group (n = 5 animals in each group). (B and D) Iron deposition/infiltration in cardiac tissue for each group was stained and quantified. Representative results of three separate experiments are shown. Bar = 200 μm; **p < 0.001 vs control; ^††^p < 0.001 vs I+G.

We investigated whether simvastatin, a common clinically used HMG-CoA reductase inhibitor, can play beneficial role in attenuating cardiac inflammation, iron deposition, or abrogating cardiac thrombosis in I+G mice. Cardiac tissue from the I+G group, and I+G plus statin (I+G+St) and the control group was collected at the end of 4^th ^week and compared. Incidence of thrombi formation were 0/10 in the control group, 7/10 in the I+G, and 2/10 in the I+G+St groups (p < 0.05 versus I+G group), respectively. Concomitant TUNEL assay and iron staining showed a significant decrease in apoptotic cardiomyoctes (Figures 5A and 5C) and iron deposition (Figures 5B and 5D) in the I+G+St compared to the I+G group.

### I+G mice shows leukocytosis and systemic elevation of inflammatory profile which can be attenuated by simvastatin but not by tirofiban treatment

**Figure 6:**
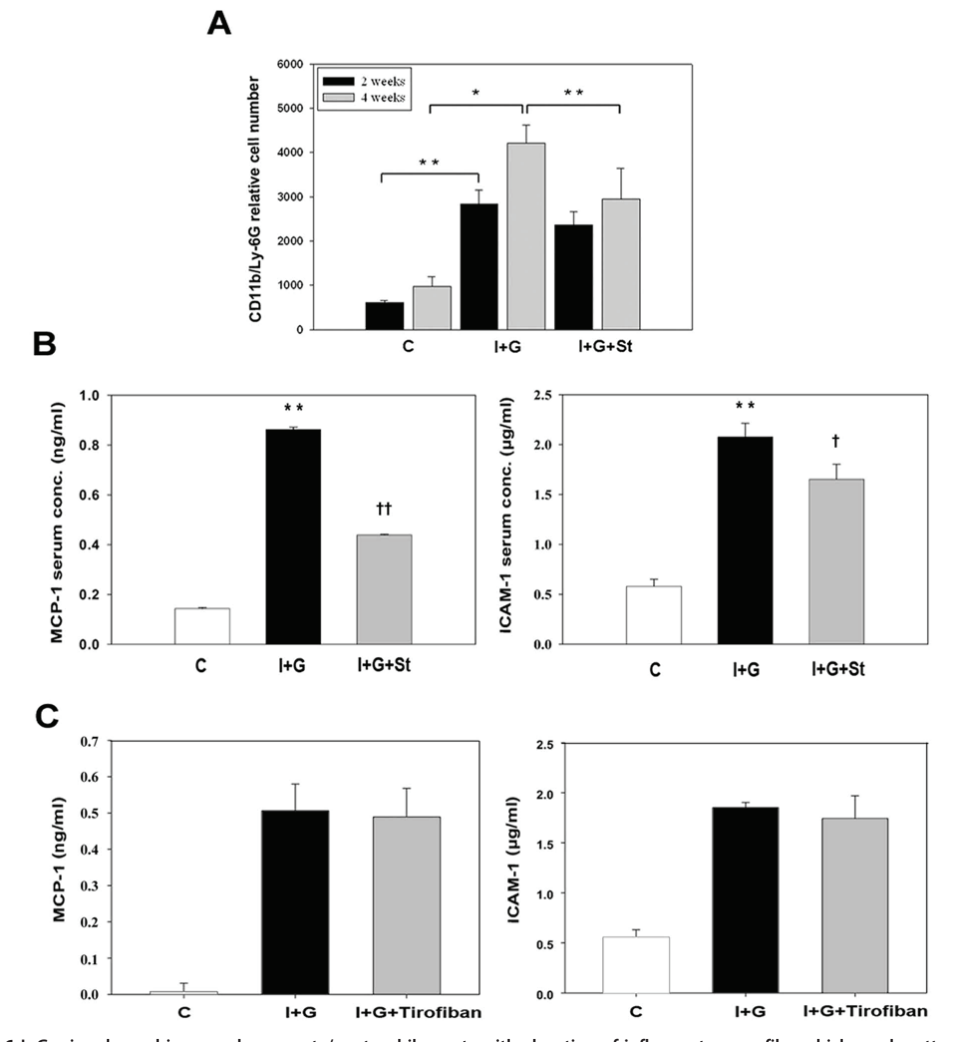
**I+G mice showed increased monocyte/neutrophil counts with elevation of inflammatory profiles which can be attenuated by simvastatin therapy, but not by tirofiban treatment**. (A) Expression of CD11b on blood serum collected from control (C), I+G, and I+G with simvastatin treatment (I+G+St) groups respectively. Blood was labeled with PE-conjugated rat anti-mouse CD11b antibody and FITC-conjugated Ly-6G monoclonal antibody separately, then flow cytommetry was performed on a BD FACScan flow cytometry system. Experiments were performed twice with similar results (n = 3 mice in each group); * p < 0.05, ** p < 0.001, respectively. (B) The mouse serum was harvested and the protein levels of MCP-1 and ICAM-1 were determined by ELISA; ** p < 0.001 vs control group; ^**† **^P < 0.05, ^**†† **^P < 0.01 vs I+G group, respectively. (C) The mouse serum was collected from control, I+G, and I+G with tirofiban treatment groups respectively.

**Figure 7:**
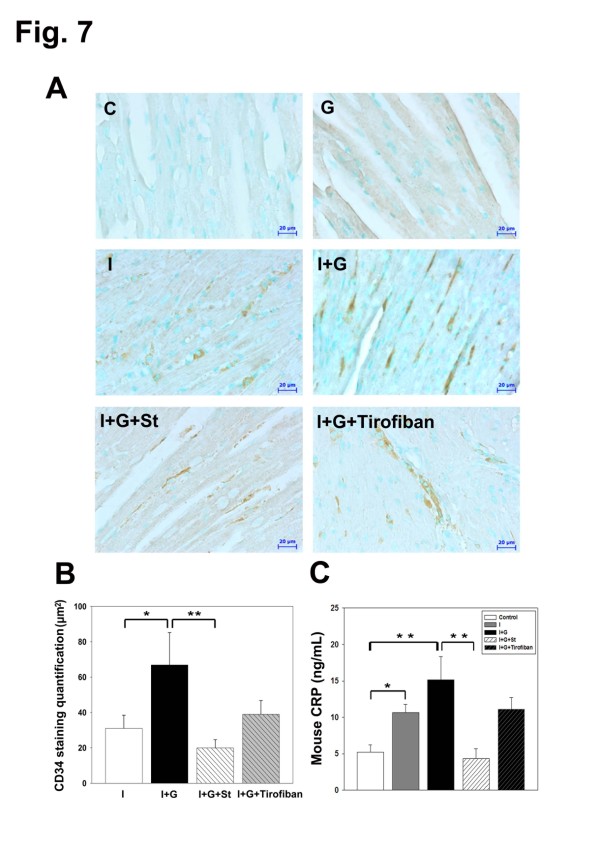
**I+G mice showed increased cardiac CD34 expression with elevation of serum c-reactive protein (CRP) levels which can be attenuated by simvastatin therapy, but not by tirofiban treatment**. (A) Immunoreactivity of CD34 were compared and quantified among heart tissue of each experimental group as indicated. Representative results of three separate experiments are shown in (B). (C) Serum CRP levels were examined via ELISA among each experimental group as indicated, *p < 0.05, ** p < 0.001.

To further determine if simvastatin act through its anti-inflammatory effect systemically, we checked complete blood counts and inflammatory profiles in the serum from I+G and I+G+St groups. Monocytes and neutorophils were increased in the serum from I+G mice at the end of second week. At the 4^th ^week recheck, leukocytosis was aggravated in the I+G mice, but attenuated in the I+G+St mice (Table 2). Flow cytometry analysis of CD11b and Ly6G proteins (myeloid cells surface markers expressed mainly on the monocytes, macrophages and granulocytes) showed increased expression in the I+G but not in the I+G+St group (Figure 6A). Serum inflammatory markers MCP-1 and ICAM-1 were up-regulated in the I+G, but not in the I+G+St group (Figure 6B). We next intended to clarify the role of platelet in this I+G induced thrombosis model, by giving platelet receptor inhibitor tirofiban to I+G mice. Interesting, although number of platelets decreased (see Additional file 1, Table S1), inflammatory profiles (Figure 6C) and thrombus formation stayed the same between I+G and I+G plus tirofiban groups (7/10 versus 7/10, respectively). Concomitant to the above results, I+G group demonstrated lower cardiac CD34 expression and serum CRP level after simvastatin therapy, but not tirofiban treatment (Figure 7). These results provide *in vivo *evidence that G-CSF-induced thrombosis can only be ameliorated by simvastatin therapy, but not by tirofiban treatment, implying a significant role of inflammation association in our model.

**Table 2:** Blood count parameters (mean ± SD) acquired at end of second and fourth weeks of I+G mice with or without statin therapy

	**LEUK (10**^**9**^**/L)**	**ERY (10**^**12**^**/L)**	HGB (g/dl)	**NEU (10**^**9**^**/L)**	**LYM (10**^**9**^**/L)**	**MONO (10**^**9**^**/L)**	**PLT (10**^**9**^**/L)**
2wks							
C	8.79 ± 1.98	8.27 ± 0.33	13.98 ± 0.61	1.44 ± 0.13	8.89 ± 1.54	0.07 ± 0.06	1330.33 ± 45.88
I	8.20 ± 3.19	9.09 ± 0.88	15.80 ± 1.18	1.21 ± 0.37	6.07 ± 2.61	0.71 ± 0.28^†^	1167.78 ± 87.37
I+G	12.07 ± 0.9*	8.36 ± 0.51	14.28 ± 0.65	2.07 ± 0.22*	7.68 ± 2.16	0.72 ± 0.07^†^	1277.33 ± 34.08
I+G+St	8.15 ± 1.77^‡^	7.53 ± 0.26	13.63 ± 1.01	2.81 ± 0.87^‡^	5.22 ± 1.23^‡^	0.62 ± 0.03	1025.25 ± 420.78

4wks							
C	9.93 ± 2.76	9.35 ± 0.28	16.08 ± 0.77	1.75 ± 0.18	6.46 ± 1.47	0.19 ± 0.02	1514.4 ± 76.51
I	19.1 ± 5.18^†^	9.36 ± 0.04	15.60 ± 0.01	11.50 ± 0.14^†^	7.39 ± 0.36	1.68 ± 0.56*	1455.2 ± 129.67
I+G	25.02 ± 2.53^†^	8.26 ± 0.27	15.46 ± 0.29	11.06 ± 1.05^†^	9.37 ± 1.59*	2.26 ± 0.32^†^	1313.8 ± 120.34*
I+G+St	18.86 ± 3.45^‡^	8.40 ± 0.26	15.7 ± 0.58	9.51 ± 0.61^‡^	5.88 ± 1.31^‡^	1.27 ± 0.59^‡^	1433.7 ± 156.18

LEUK, leukocytes; ERY, erythrocytes; HGB, hemoglobin; NEU, neutrophil; LYM, lymphocyte; MONO, monocyte; PLT, platelet; *p < 0.05, ^†^p < 0.01 vs control; ^‡ ^p < 0.05 vs I+G, n = 8 in each group.

### Simvastatin also ameliorates inflammatory stage in the heart tissue of I + G mice

**Figure 8:**
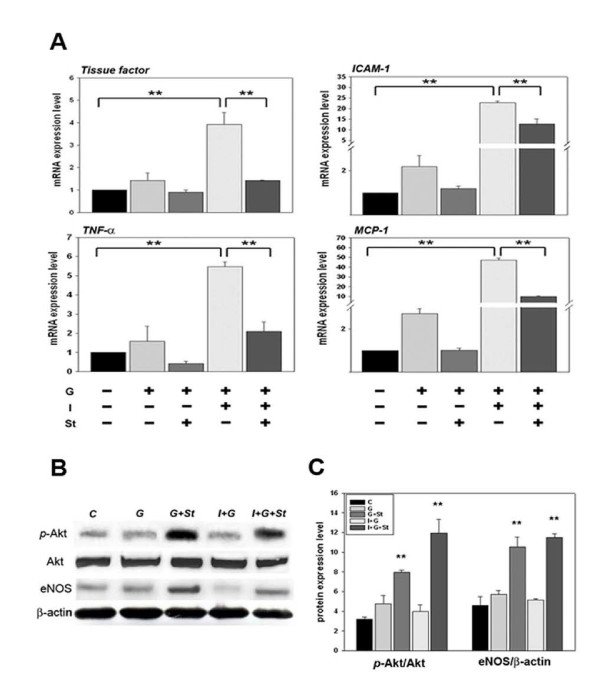
**Cardiac mRNA analysis for inflammatory markers and protein analysis for AKT and eNOS expression in I+G mice compared with I+G plus simvastatin treated mice**. (A) Total mRNAs were prepared from whole heart tissues, and the levels of ICAM-1, MCP-1, tissue factor, and TNF-alpha transcripts were determined by Quantitative-PCR analysis. Note that the levels of four transcripts, especially of tissue factor and TNF-alpha reduced significantly after simvastatin administration. GADPH expression was used as a control to monitor RNA quality and concentration; **p < 0.001. (B and C) Western blot analysis of phosphorylated AKT (pAkt), AKT, eNOS, and β-actin. Lanes from left to right indicate heart tissues taken from the untreated control (C), G-CSF only (G), G-CSF with statin administration (G+St), I+G, and I+G with simvastatin administration (I+G+St). Data represent results from three independent experiments. Scanning densitometry was used for semi-quantitative analysis in compared to the Akt or β-actin levels respectively; **p < 0.001 vs control.

Heart tissue was sampled at the end of 4^th ^week for quantitative PCR analysis. Expression of ICAM-1, MCP-1, TNF-α, and tissue factor increased in the I+G group compared with the control group (Figure 8A). Interestingly, increased expression of MCP-1 and ICAM-1 were also noted in the G-group (p < 0.05 versus control), indicating that G-CSF alone can promote pro-inflammatory factors. Decreased expression of the above pro-inflammatory factors was seen in the I+G+st group (Figure 8A). This result suggested that simvastatin attenuated the cardiac thrombus formation via down regulation of inflammatory signaling in the heart tissue.

### Elevated pAkt and eNOS expression in simvastatin supplemented hearts

To elucidate the molecular pathway of statin's anti-inflammation therapy on I+G mice. Protein levels of phosphorylated Akt (pAkt) and endothelial nitric oxide synthase (eNOS) increased in the hearts of the G plus statin and I+G+St groups, as compared to other groups (Figure 8B). These results indicate that statin treatment significantly enhanced the expression of eNOS and phosphorylation of Akt, and that the therapeutic effect of statin in ameliorating the thrombus formation may act through the activation of Akt-eNOS signaling pathway.

## Discussion

Results of the present study demonstrate that G-CSF supplement on iron loading hearts can recruit neutrophils/monocytes and up-regulate tissue factors, ICAM-1, TNF-alpha, and MCP-1 thus further activating inflammatory processes in the endo-myocardium and induce cardiac thrombosis. Chronic iron loading can increase cardiac oxidative stress. Whereas G-CSF treatment activates serial events of inflammation-thrombosis circuitry and that leads to intra-cardiac thrombus formation. This inflammation-associated cardiac thrombosis *in vivo *can be attenuated by simvastatin therapy, but not by tirofiban treatment. Our results confirmed that G-CSF can induce *in vivo *cardiac thrombosis through inflammation-thrombosis interaction.

Iron overload is known to accelerate arterial thrombosis through increased vascular oxidative stress and impaired vascular reactivity [16,21] and it also impairs cardiac function by increasing free radical production resulting in cardiomyopathy [22,23]. However, present study shows that iron loading alone is not sufficient to induce intra-cardiac thrombosis as reported by others [20]. Our results clearly indicate that G-CSF supplementation effectively initiated inflammation-thrombosis bridging thereby promoting thrombosis and recruited subsets of hematopoietic cells, like mature neutrophils and monocytes which bear their adhesion receptors on the cell membrane [24]. Moreover, recent reviews also reported a pivotal role of tissue factor in driving the thrombosis- inflammation circuit [25,26]. This may be responsible for accumulation of a large number of macrophages and tissue factor expression in the affected lesions (Figure 3B). G-CSF induced leukocyte infiltration resulted in increased tissue factor expression with secondary thrombosis and subsequent tissue fibrosis. As tirofiban fail to ameliorate the thrombosis, it may indicate that fibrinogen (or GPIIb/IIIa) did not have major role in this inflammation-thrombosis process [27]. Our *in vivo *mouse model could be a novel avenue for investigating inflammation and thrombosis interactions in the cardiac endothelium, compared to previous studies that focused mainly on the vascular endothelium [27,28].

Iron loading has multiple effects on all body tissues, including cardiac myocytes and macrophages. For example, in a similar iron overload model (with chronic iron treatment for 12 weeks) showed increased cardiac interstitial fibrosis in addition to inflammatory infiltration [19]. Iron-overloaded macrophage secrete increased levels of cytokines in response to an inflammatory stimulus and exacerbates alcoholic liver injury [29,30]. In our I+G model, G-CSF supplementation increased ROS production and recruitment of leukocyte (Figure 4) further aggravated inflammatory infiltration which eventually triggered cardiac thrombosis. However, thrombosis only seen in the cardiac chamber but not other organs (see Supplementary Figure 1), may be due to the fact that macrophage are prone to be deposited in the heart and the liver, yet the latter organ lacks the shear stress induced by rapid blood flow and functional impaired endothelium unlike the heart.

Our results showing that G-CSF can promote inflammatory profiles and cardiac thrombosis that leads to cardiac dysfunction, are in contrast to previous reports showing G-CSF therapy to be beneficial in acute myocardial infarction [3,4,31,32] and chronic cardiomyopathy induced by doxorubicin toxicity [33]. G-CSF exerts an anti-inflammatory effect [34] as well as an angiogenic and anti-apoptotic effect which prevents LV wall thinning and heart failure after acute myocardial infarction [3,35]. One explanation for these disparate results could be that chronic iron loading increases oxidative stress and impairs endothelium-dependent vaso-relaxation [16], a different scenario than in acute myocardial infarction. Although G-CSF recruits hematogenic stem cells and endothelial progenitor cells for cardiac repair, a simultaneous induction of macrophage and tissue factor gathering "gears up" the pro-inflammatory state and drives the inflammation-thrombosis circuit. Besides, G-CSF induced leukocytosis is a well known feature that also suggests its direct role in enhancing acute thrombosis [36].

HMG-CoA reductase inhibitors, or statins, are known to improve cardiac dysfunction through their anti-inflammatory and anti-oxidative action. Statins also affect endothelial function through the production of nitric oxide [18,19]. Present study demonstrates that simvastatin can reduce the myocardial iron deposition/infiltration score (Figure 4D) and blood leukocyte count (Table 2) that strengthens the link between inflammation and myocardial thrombus formation. Simvastatin administration significantly reduced the incidence of thrombus formation in the I+G heart, and expression of the pro-inflammatory markers ICAM-1, tissue factor, MCP-1, and TNF-α. Furthermore, prior studies suggesting that statin could regulate eNOS activity via post-translational activation of phosphatidylinositol 3-kinase/protein kinase Akt pathway (PI3K/Akt) in the endothelium [37-40]. Simvastatin treated I+G hearts in our study revealed an elevation of both eNOS and phosphorylated Akt activity, suggesting that simvastatin had a therapeutic effect in ameliorating the thrombus formation in the heart.

Recently meta-analysis results from 10 clinical trials for stem cell mobilization by G-CSF therapy for myocardial recovery after AMI showed neither improvement of LV function or the reduction in infarct size in patients with AMI after reperfusion [8]. In order to effectively improve LV contractility, future studies should focus more on the autologous stem cells plus G-CSF infusion. Under such scenario, more attention should be paid to the possible detrimental effects of G-CSF related thrombosis. As G-CSF plus stem cells might additively increase cell density and hypercoagulable state in certain time window thus result in re-stenosis or late thrombosis in MI patients. Therefore, it is important to screen for high risk patients with chronic inflammation or increased oxidative stress like metabolic syndrome, diabetes, chronic heart failure, or chronic atherosclerosis, before they should receive G-CSF treatment for acute coronary heart disease. Accordingly, present study provides an *in vivo *disease model to elucidate the mechanism of post G-CSF cardiac thrombosis, which could have major clinical implication.

## Competing interests

The authors declare that they have no competing interests.

## Authors' contributions

WSL and CFC designed the experiments and analyzed the data. WSL and HL performed the in vivo study. HL performed the in vitro study. TK analyzed the cardiac pathology. WTKC and TK help to coordinate this study. CFC wrote the manuscript. All authors have read and approved the final manuscript.

## Supplementary Material

Additional file 1 **Histology of I+G mice and blood parameters of I+G mice with tirofiban treatment**. A figure demonstrating histology of other organs in I+G mice and a table listing blood parameters of I+G mice with or without tirofiban therapy. Click here for file
