# Supplementary material for: Granulocyte-CSF induced inflammation-associated cardiac thrombosis in iron loading mouse heart and can be attenuated by statin therapy
Source: J Biomed Sci. 2011 Apr 15;18(1):26. doi: 10.1186/1423-0127-18-26 (PMC3095536; doi:10.1186/1423-0127-18-26)
Supplement: Additional file 1 — Histology of I+G mice and blood parameters of I+G mice with tirofiban treatment. A figure demonstrating histology of other organs in I+G mice and a table listing blood parameters of I+G mice with or without tirofiban therapy. [file 1423-0127-18-26-S1.DOC]

**Figure S1. Histology of other organs in I+G mice.**

(A). Prussian blue staining was used to demonstrate iron infiltration/ deposition in aorta, liver, and kidney, respectively. (B) H & E staining was used demonstrate that no thrombi formation was found in either coronary artery (arrow) or liver in I+G mice. Iron particles (brown color) were accumulated in macrophages infiltrating into connective tissue space around the coronary artery, and iron particles were also found within cytoplasm of many liver cells.

**
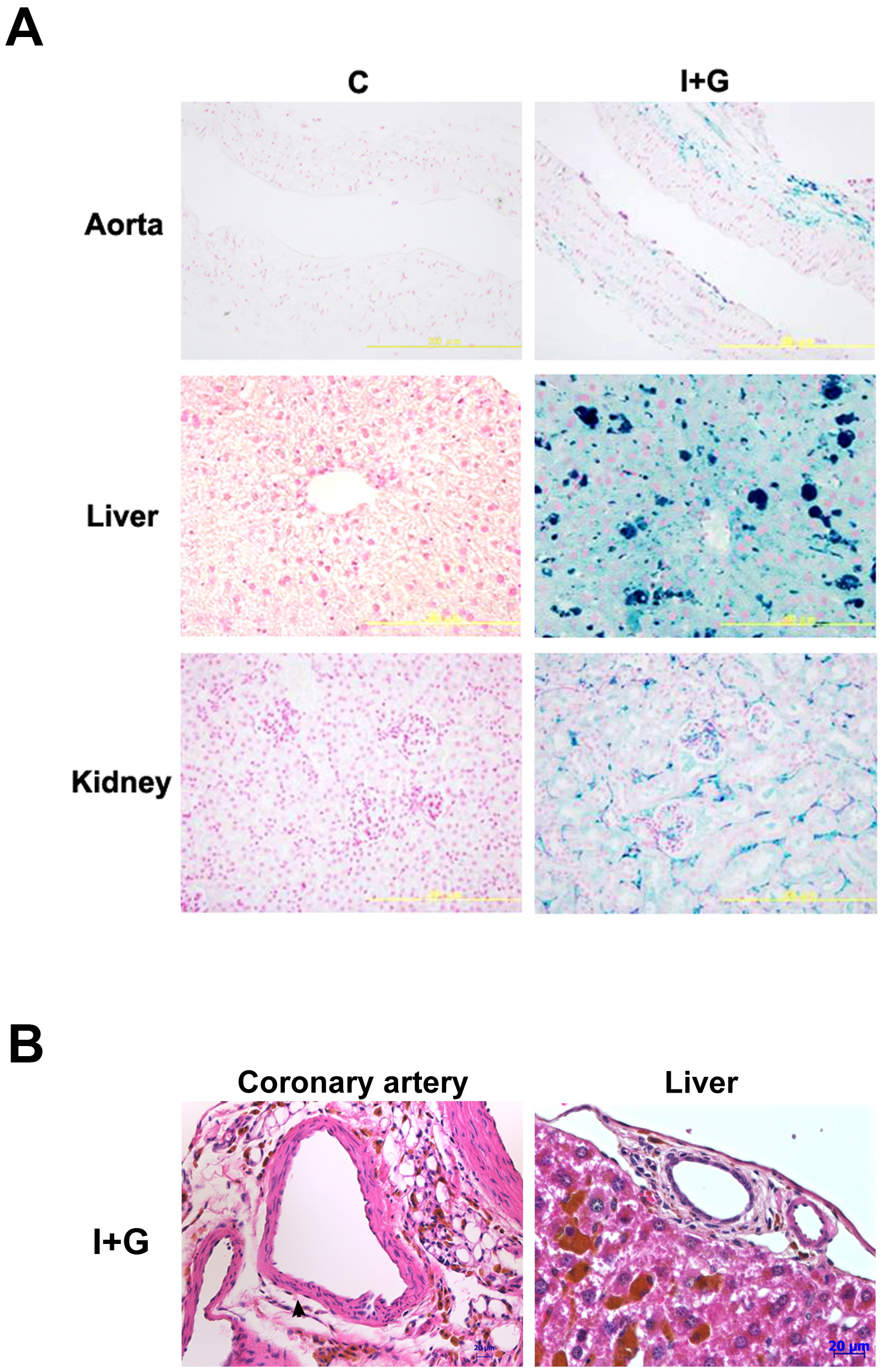
**

**Table S1. Blood count parameters (mean + SD) acquired at end of second week of I+G mice with or without tirofiban treatment**

|  | **LEUK(10^9^/L)** | **ERYTH (10^12^/L)** | **HGB(g/dl)** | **NEU(10^9^/L)** | **LYM(10^9^/L)** | **MONO(10^9^/L)** | **PLT(10^9^/L)** |
| --- | --- | --- | --- | --- | --- | --- | --- |
| **2wks** |  |  |  |  |  |  |  |
| **C** | **8.36±0.97** | **11.46±0.38** | **17.04±0.94** | **1.25±0.14** | **6.84±1.02** | **0.08±0.06** | **1062.6±34.3** |
| **I+G** | **17.7±2.46^†^** | **10.50±0.36** | **15.80±0.30** | **9.49±0.95^†^** | **8.36±1.65*** | **0.66±0.14^†^** | **1233.9±31.1** |
| **I+G+Tirofiban** | **20.7±4.45^‡^** | **11.15±1.06** | **16.55±1.34** | **8.02±2.15** | **5.14±0.97^‡^** | **0.90±0.60^‡^** | **905.9±115.0^‡^** |
|  |  |  |  |  |  |  |  |

LEUK, leukocytes; ERYTH, erythrocytes; HGB, hemoglobin; NEU, neutrophil; LYM, lymphocyte; MONO, monocyte; PLT, platelet; *p<0.05, ^†^p<0.01 vs control; ^‡^ p<0.05 vs I+G, n=8-10 in each group.
